# Influence of Hypoxic Interval Training and Hyperoxic Recovery on Muscle Activation and Oxygenation in Connection with Double-Poling Exercise

**DOI:** 10.1371/journal.pone.0140616

**Published:** 2015-10-15

**Authors:** Christoph Zinner, Anna Hauser, Dennis-Peter Born, Jon P. Wehrlin, Hans-Christer Holmberg, Billy Sperlich

**Affiliations:** 1 Department of Sport Science, Julius-Maximilians-University Würzburg, Würzburg, Germany; 2 Swedish Winter Sports Research Centre, Department of Health Sciences, Mid Sweden University, Östersund, Sweden; 3 Swiss Federal Institute of Sport, Section for Elite Sport, Magglingen, Switzerland; Norwegian University of Science and Technology, NORWAY

## Abstract

Here, we evaluated the influence of breathing oxygen at different partial pressures during recovery from exercise on performance at sea-level and a simulated altitude of 1800 m, as reflected in activation of different upper body muscles, and oxygenation of the *m*. *triceps brachii*. Ten well-trained, male endurance athletes (25.3±4.1 yrs; 179.2±4.5 cm; 74.2±3.4 kg) performed four test trials, each involving three 3-min sessions on a double-poling ergometer with 3-min intervals of recovery. One trial was conducted entirely under normoxic (No) and another under hypoxic conditions (Ho; F_i_O_2_ = 0.165). In the third and fourth trials, the exercise was performed in normoxia and hypoxia, respectively, with hyperoxic recovery (HOX; F_i_O_2_ = 1.00) in both cases. Arterial hemoglobin saturation was higher under the two HOX conditions than without HOX (p<0.05). Integrated muscle electrical activity was not influenced by the oxygen content (best d = 0.51). Furthermore, the only difference in tissue saturation index measured via near-infrared spectroscopy observed was between the recovery periods during the NoNo and HoHOX interventions (*P*<0.05, d = 0.93). In the case of HoHo the athletes’ P_mean_ declined from the first to the third interval (P < 0.05), whereas P_mean_ was unaltered under the HoHOX, NoHOX and NoNo conditions. We conclude that the less pronounced decline in P_mean_ during 3 x 3-min double-poling sprints in normoxia and hypoxia with hyperoxic recovery is not related to changes in muscle activity or oxygenation. Moreover, we conclude that hyperoxia (F_i_O_2_ = 1.00) used in conjunction with hypoxic or normoxic work intervals may serve as an effective aid when inhaled during the subsequent recovery intervals.

## Introduction

The influence of inhaling different partial pressures of oxygen (i.e., hypoxia and hyperoxia) on the activity and oxygenation of various muscle groups at different exercise intensities has been characterized [1–3]. Oxygen (O_2_) availability has a crucial impact on the time required for recovery of phosphocreatine, as well as on the diffusion of O_2_ into muscle cells [4–6]. With more and more pronounced hypoxia, the changes in arterial oxygen saturation (S_a_O_2_) evoke cardio-respiratory efforts designed to compensate for the reduction in systemic O_2_ transport and counteract impairment of performance and muscle activity [7]. At the same time, breathing hyperoxic air elevates the amount of oxygen dissolved in the arterial plasma [8, 9], thereby potentially accelerating recovery. In recent years, the physiological responses of athletes to high intensity exercise with intermittent hypoxic training as well as repeated sprints in hypoxia, have attracted increasing scientific interest [10–12].

Recent findings regarding the effects of hypoxia on muscle oxygenation during high-intensity exercise, mainly involving lower-body muscles, have been inconsistent. For example, during cycle sprints, oxygenation of the *vastus lateralis* muscle has been reported to be the same in hypoxia as normoxia [13], whereas during running sprints hypoxia aggravated muscle deoxygenation [1]. Furthermore, moderate hyperoxia (F_i_O_2_ = 0.30) exerted no effects on the kinetics of muscle deoxygenation at the onset of heavy exercise [14].

During high-intensity exercise, the maximal force produced, rates of force production and relaxation [15], and recruitment of motor units and their rate of firing (as reflected in the integrated muscle electrical activity (iEMG)) are all reduced [16]. In contrast, during prolonged submaximal exercise to exhaustion, iEMG rises [17]. The electrical signals of exercising skeletal muscles are affected by alterations in the oxygen content of air (F_i_O_2_), with elevations in F_i_O_2_ enhancing muscle activity and power output [8, 18, 19]. On the other hand, in comparison to normoxia, hypoxia does not alter muscle activity during whole-body exercise at the same relative exercise intensities, but does elevate this activity during exercise of similar absolute intensity [20]. Hyperoxia can influence power output during repeated high-intensity exercise, and the role of neuromuscular factors in this context is of considerable interest.

Along with impaired neuromuscular function and intracellular perturbations (e.g., accumulation of inorganic phosphate and alterations in pH) [11, 21], the level of oxygen supply to skeletal muscles, regulated by blood flow and vasodilation [22], is a key determinant of fatigue during high-intensity exercise. Near-infrared spectroscopy (NIRS) has been employed to evaluate the level of oxygenation in human skeletal muscle [23], potentially reflecting the balance between oxygen supply and utilization [24, 25]. Recently, muscle oxygenation during periods of recovery between sprints, which may be an indicator of the ability to perform high-intensity repetitions effectively, has been examined [26, 27].

However, little information is presently available regarding acute alterations in muscle activity and oxygenation during and after high-intensity during whole-body exercise under hypoxic or hyperoxic conditions [12]. To the best of our knowledge, the influence of hypoxia and hyperoxia on oxygenation of the arm muscles during high-intensity exercise has not yet been examined. Since these muscles perform most of the work during double poling [28], the influence of different F_i_O_2_ during recovery on double poling performance is of considerable interest.

International competitions (e.g., in cross-country skiing) are held at altitudes ranging from near sea-level to above 2000 m. Accordingly, the primary goal of the present investigation was to evaluate the influence of breathing different partial pressures of oxygen during recovery from skiing at either sea-level or a simulated altitude of 1800 m on activation of the *m*. *biceps brachii*, *triceps brachii lateralis*, *latissimus dorsi*, and *pectoralis major* and oxygenation of the *m*. *triceps brachii*. We hypothesized that both tissue oxygen saturation and muscle activity during intervals of exercise differ with hypoxic and hyperoxic recovery.

## Materials and Methods

### Participants and Ethical Statement

The 10 well-trained, male endurance athletes (25.3 ± 4.1 years, 179 ± 4.5 cm, 74.2 ± 3.4 kg; fat mass: 9.2 ± 2.9%; VO_2_max: 62.0 ± 7.2 mL·kg^-1^·min^-1^, (means ± SD)) who volunteered to participate were all familiar with the laboratory exercise procedures employed here. They were asked to report for the tests in an adequately hydrated condition and to refrain from consuming alcohol for 24 h and food or caffeine for 3 h prior to each test. Before participating, all athletes were informed of the protocol and provided their written consent. All procedures were pre-approved by the ethics committee of the German Sport University in Cologne and conducted in accordance with the Declaration of Helsinki.

These participants were recruited from local clubs whose members are involved in endurance sports. All were well-trained cross-country skiers and triathletes, with well-trained upper-bodies, and cross-country and roller skiing were included in their regular training. Moreover, each had trained for at least 5 years. Prior to actual testing, all performed at least 5 training sessions for familiarization with the double-poling (DP) ergometer and protocols. In connection with the last of these training sessions, body composition was determined by 4-electrode bioimpedance (Tanita BC 418 MA, Tanita Corp, Tokyo, Japan).

### Design and Procedures

Within a 3-week period, all of the participants completed 4 trials in random, counterbalanced order, separated by at least 3 days to guarantee adequate recovery. Each trial was composed of three 3-min sessions of double-poling and subsequent 3-min recovery. Each trial was performed as follows: a) normoxic (fractional content of oxygen, F_i_O_2_ = 0.21) exercise with normoxic recovery (NoNo); b) normoxic exercise with hyperoxic (F_i_O_2_ = 1.00) recovery (NoHOX); c) hypoxic (F_i_O_2_ = 0.165) exercise with hypoxic recovery (HoHo); or d) hypoxic exercise with hyperoxic recovery (HoHOX).

To achieve hypoxia, the athlete inhaled air through a face mask connected with plastic tubing to an AltiTrainer200® (SMTEC, Nyon, Switzerland), which mixes ambient air with nitrogen to obtain the desired partial pressure of inspired oxygen. To ensure accuracy, this apparatus was calibrated at the beginning of each test day, taking into account the actual barometric pressure. During the recovery periods, hyperoxic air was delivered from a 170 L Douglas Bag (Hans Rudolph Inc., Shawnee, KS, USA), again attached to a face mask with plastic tubing. Each participant wore the same face mask and breathed through the same tubing during all 4 interventions. Here, a single-blinded study design was employed, with the athletes having no knowledge of what type of air they were breathing and not being allowed to see the device supplying this air. The transition between the different F_i_O_2_ was ensured by a switch valve and was immediately changed after each interval/recovery period.

During the three 3-min simulated DP sprints on a cross-country ski ergometer (SkiErg, Concept2, Hamburg, Germany), each athlete selected his own pacing strategy designed to ensure maximal mean power output and the power output during each poling cycle was recorded. Pilot testing of three subjects revealed coefficients of variation for repeated measurements of the mean power output ranging from 4.9–6.3%. During the 3-min period of recovery between sprints, the subjects walked (1.0 m/s) on a treadmill beside the ergometer. The transition from the DP ergometer to the treadmill and the start of the walk took approximately 30 s.

EMG signals from the surfaces of the *biceps brachii*, *triceps brachii lateralis*, *latissimus dorsi*, and *pectoralis major* muscles were recorded with a Noraxon TeleMyo 2400 T G2TM telemetered system (Noraxon Inc., Scottsdale, AZ, USA). For this purpose, bipolar electrodes pre-coated with gel were placed on the bellies of these muscles, in alignment with the underlying muscle fibers and in accordance with international standards [29]. The grounding electrode was positioned on the acromion. The distance between the centers of the two electrodes on each muscle was 2 cm. Prior to positioning the electrodes, the surface of the skin was shaved, abraded slightly, and cleansed with alcohol.

All EMG signals were recorded at a sampling frequency of 2000 Hz using a differential amplifier (Biovision, Wehrheim, Germany) and a 10-500-Hz band-pass filter at 3 dB. The data thus obtained were converted to digital units (DAQ 700 A/D card, National Instruments, Austin, TX, USA) and analyzed using the MyoResearch software, version 1.06.50 (Noraxon Inc., Scottsdale, AZ, USA). EMG values were calculated using the moving-window technique (stepwise value-for-value; window size, 250 ms). Prior to further processing, all of the EMG signals were full-wave rectified and the root mean square of the value for each 250-ms window calculated.

During all testing, oxygenation of the medial portion of the *m*. *triceps brachii* was measured by near-infrared spectroscopy (NIRS) (Portamon, Artinis Medical Systems, Zetten, The Netherlands). The distance from the elbow (along the vertical axis of the upper arm) to the center of this device was approximately 12 cm and the thickness of the skin and subcutaneous tissue (as determined with Harpenden calipers; British Indicators Ltd, West Sussex, UK) at the site of measurement was less than half the distance between the source and detector. To optimize standardization of the localization of the NIRS device and EMG electrodes, the same individual prepared all of the subjects. Additionally, a marker on the skin allowed accurate repositioning of the sensor for each subsequent trial. Alterations in tissue concentrations of oxy- [HbO_2_], deoxy- [HHb], and total hemoglobin [tHb] were monitored at wavelengths of 760 and 840 nm. The tissue saturation index (TSI) (calculated as [HbO_2_]/([HbO_2_] + [HHb])×100 and expressed as %) was used as an indicator of the balance between oxygen supply and consumption. Unfortunately, it was not possible to measure more muscles with NIRS, since only one device was available.

**Fig 1:**
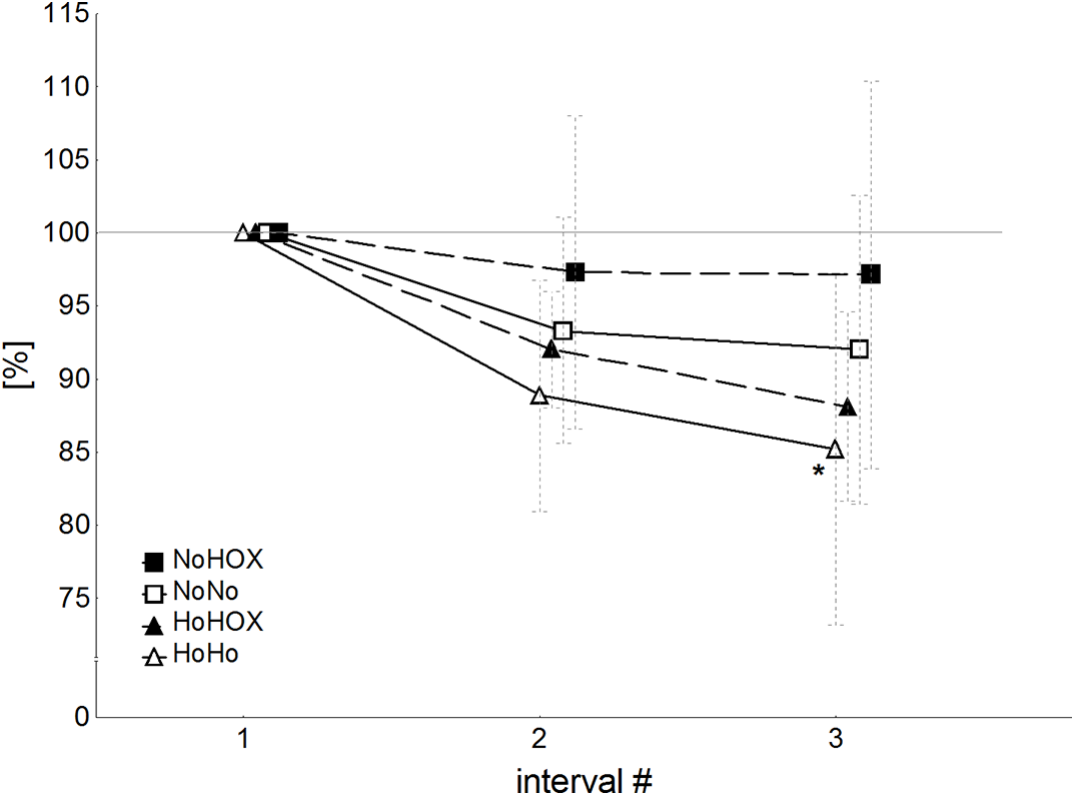
Mean power output during the three intervals under all four conditions. * indicates *P*<0.05 in comparison to interval #1. NoNo: normoxia with normoxic recovery; NoHOX: normoxia with hyperoxic recovery; HoHo: hypoxia with hypoxic recovery; HoHOX: hypoxia with hyperoxic recovery.

For analysis of the lactate concentration, 20 μL blood was collected from the left earlobe into a capillary tube (Eppendorf AG, Hamburg, Germany) both before and after the 10-min warm-up period, as well as immediately and 2.5 min after the 3-min DP sprints (Fig 1). For analyses of pH and blood gases (PO_2_, PCO_2_, SO_2_), 120 μL capillary blood was collected from the right earlobe at these same time-points, when the participants were also asked to rate their perceived exertion on the 6–20-point Borg scale. Blood lactate was assayed with an amperometric-enzymatic procedure using the Ebio Plus system (Eppendorf AG, Hamburg, Germany) and blood gases with the AVL Omni 3 system (Roche Ltd, Basel, Switzerland), in duplicate in all cases, with the mean being used for statistical analysis. Under our laboratory conditions, the coefficient of variation for repeated measurements of lactate concentration is routinely 1.2% at a concentration of 12 mmol/L. For arterial oxygen saturation and partial pressure, the corresponding coefficients of variation are 3.2 and 3.6%, respectively.

### Statistical analyses

All data analysis involved parametric procedures and mean values and standard deviations (SD) are presented. All variables demonstrated a normal distribution, so that no further transformation was required. All parameters under the four different interventions were compared using a 2-factor [intervention (NoNo, NoHOX, HoHo, HoHOX); time (baseline, post-WU, 0´ and 2:30 min post-1^st^, -2^nd^ and -3^rd^] repeated-measures ANOVA with Tukey post-hoc analysis, where P < 0.05 was considered significant. All analyses were carried out with the Statistica software package for Windows® (version 7.1, StatSoft Inc., Tulsa, OK, USA). The effect size Cohen's d (defined as the difference between the means divided by the standard deviation [30]) was calculated and the thresholds for small, moderate, and large effects defined *a priori* as 0.20, 0.50, and 0.80, respectively [30].

## Results

**Fig 2:**
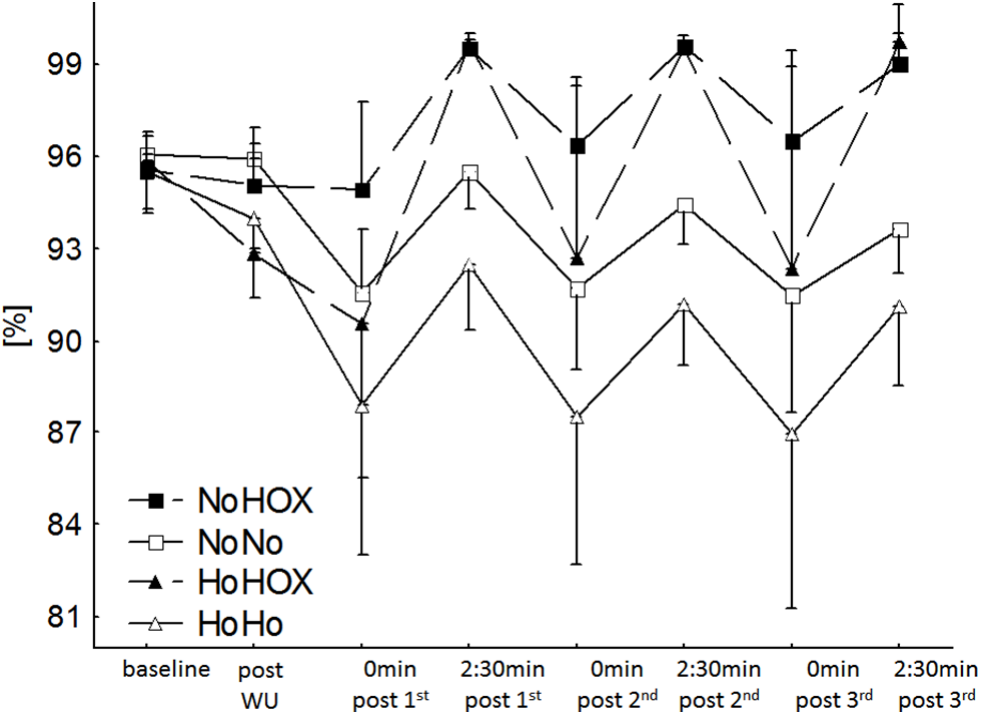
Changes in arterial oxygen saturation (S_a_O_2_) during the three intervals under all four conditions. For clarity, the associated statistical analyses are documented in Table 2. NoNo: normoxia with normoxic recovery; NoHOX: normoxia with hyperoxic recovery; HoHo: hypoxia with hypoxic recovery; HoHOX: hypoxia with hyperoxic recovery.

While double-poling for 3 min under either hypoxic or normoxic conditions with hyperoxic recovery, S_a_O_2_ was significantly higher than at baseline or during the other two trials (Table 1 and Fig 2) (d = 4.65). The PO_2_ was increased approximately 2–3.5-fold at the end of the HOX recovery periods, being significantly higher than with normoxic or hypoxic recovery (d = 5.71).

**Table 1:** The values (means ± SD) of the different metabolic and perceptual responses of the athletes (n = 10) during the 3x3-min DP intervals in association with the different oxygen contents during the intervals and recovery periods. NoNo: normoxia with normoxic recovery; NoHOX: normoxia with hyperoxic recovery; HoHo: hypoxia with hypoxic recovery; HoHOX: hypoxia with hyperoxic recovery.

		baseline	post WU	0´ min post 1^st^	2:30 min post 1^st^	0´ min post 2^nd^	2:30 min post 2^nd^	0´ min post 3^rd^	2:30 min post 3^rd^
**S**_**a**_ **O**_**2**_ **[%]**	**HoHo**	95.5±1.4	94.0±1.0	87.9±4.9^a^ ^d^	92.5±2.2d	87.5±4.8^a^ ^c^ ^d^	91.2±2.0^a^ ^c^ ^d^	87.0±5.7^a^ ^c^ ^d^	91.1±2.6^a^ ^c^
	**HoHOX**	95.8±0.8	92.9±1.5^d^	90.6±5.1^a^	99.6±0.4^a^ ^b^ ^d^	92.7±5.6^a^ ^b^	99.5±0.4^a^ ^b^ ^d^	92.3±5.6^a^ ^b^	99.7±0.3^a^ ^b^ ^d^
	**NoNo**	96.1±0.7	95.9±1.0^c^	91.6±2.0^a^ ^b^	95.5±1.2^c^	91.7±2.7^a^ ^b^	94.4±1.3^b^ ^c^	91.5±2.8^a^ ^b^	93.6±1.4^c^
	**NoHOX**	95.5±1.2	95.0±1.4	94.9±2.9^b^ ^c^ ^d^	99.5±0.3^a^ ^b^ ^d^	96.3±2.3^b^ ^c^ ^d^	99.6±0.3^a^ ^b^ ^d^	96.5±2.9^b^ ^c^ ^d^	99.0±1.9^a^ ^b^ ^d^
**PO**_**2**_ **[mmHg]**	**HoHo**	80.3±7.8	75.2±4.1	65.0±8.7	83.0±6.5^c^	69.2±7.2	81.4±2.5^c^	71.0±8.7	78.7±4.4^c^
	**HoHOX**	81.9±5.2	71.7±5.9	76.0±19.7	293.7±84.5^a^ ^b^ ^d^	98.7±32.1	302.1±52.4^a^ ^b^ ^d^	90.1±21.6	331.6±62.5^a^ ^b^ ^d^
	**NoNo**	82.4±5.2	85.4±7.6	73.7±9.9	96.2±9.5^c^	80.9±8.1	93.0±7.3^c^	79.5±8.3	89.3±4.5^c^
	**NoHOX**	81.8±7.7	82.3±7.8	104±42.1	260±98.9^a^ ^b^ ^d^	122±43.6^b^	256±62.5^a^ ^b^ ^d^	137±50.4^a^ ^b^ ^d^	254±90.7^a^ ^b^ ^c^ ^d^
**PCO**_**2**_ **[mmHg]**	**HoHo**	40.6±1.4	37.9±2.0	37.3±3.3	35.5±2.2	31.7±3.5	32.2±1.6	30.4±2.2	30.1±3.7
	**HoHOX**	40.8±1.9	39.4±2.7	38.9±4.2	36.4±2.9	33.2±3.6	32.8±3.8	31.4±3.3	32.4±2.1
	**NoNo**	40.3±1.8	38.4±1.7	38.5±2.2	35.1±.18	33.2±2.3	32.1±1.5	31.1±1.9	31.2±2.0
	**NoHOX**	41.3±3.7	39.5±2.7	38.5±2.8	36.6±.2.9	33.8±3.9	31.9±4.8	32.4±3.0	31.8±2.8
**Lactate [mmol·L** ^**-1**^ **]**	**HoHo**	0.9±0.2	3.4±1.5^a^	8.4±1.3^a^	9.7±1.9^a^	12.0±2.0^a^	12.2±2.4^a^	13.5±2.5^a^	12.3±2.9^a^
	**HoHOX**	0.9±0.2	3.4±1.8^a^	8.3±2.2^a^	9.2±2.5^a^	11.2±2.6^a^	11.7±2.6^a^	12.5±2.4^a^	11.9±2.4^a^
	**NoNo**	1.0±0.6	2.7±1.0^a^	8.3±2.4^a^	9.1±2.1^a^	11.6±2.2^a^	11.5±2.0^a^	12.9±2.3^a^	12.2±2.5^a^
	**NoHOX**	0.9±0.4	3.2±1.2^a^	7.3±1.1^a^	8.2±1.4^a^ ^b^	9.9±2.2^a^ ^b^ ^d^	10.4±2.6^a^ ^b^	11.8±2.7^a^ ^b^	11.7±3.1^a^
**pH**	**HoHo**	7.40±0.02	7.37±0.03	7.28±0.05^a^	7.25±0.05^a^	7.23±0.05^a^ ^c^	7.21±0.06^a^	7.20±0.07^a^	7.22±0.07^a^
	**HoHOX**	7.39±0.02	7.35±0.04^a^	7.25±0.07^a^	7.22±0.07^a^ ^d^	7.19±0.09^a^ ^b^ ^d^	7.18±0.08^a^ ^d^	7.19±0.08^a^	7.19±0.08^a^
	**NoNo**	7.41±0.02	7.38±0.02	7.28±0.05^a^	7.26±0.06^a^ ^c^	7.23±0.06^a^ ^c^	7.22±0.07^a^ ^c^	7.21±0.07^a^	7.20±0.07^a^
	**NoHOX**	7.40±0.02	7.35±0.03^a^	7.27±0.04^a^ ^c^	7.26±0.05^a^ ^c^	7.24±0.07^a^ ^c^	7.23±0.09^a^	7.20±0.08^a^	7.20±0.08^a^
**RPE**	**HoHo**		9.6±2.0	18.0±1.2^a^	12.6±1.2^a^	19.0±0.8^a^	13.4±1.3^a^	19.6±0.7^a^	11.8±2.1^a^
	**HoHOX**		10.0±2.4	18.2±1.8^a^	12.5±2.0^a^	19.0±1.2^a^	12.9±1.9^a^	19.4±0.7^a^	12.3±1.8^a^
	**NoNo**		9.1±2.3	17.4±1.6^a^	12.2±2.1^a^	18.4±1.4^a^	12.8±1.4^a^	19.1±1.3^a^	11.3±2.0^a^
	**NoHOX**		9.7±2.9	17.7±1.7^a^	13.1±1.6^a^	18.6±1.3^a^	13.5±1.6^a^	19.7±0.7^a^	12.3±2.0^a^ ^d^

^a^ indicates significant difference to baseline

^b^ indicates significant difference to HoHo at the same time point

^c^ indicates significant difference to HoHOX at the same time point

^d^ indicates significant difference to NoNo at the same time point

The different levels of oxygen did not influence the activity of any of the muscles monitored (best d = 0.51). Furthermore, the only difference in the level of oxygenation (TSI) during the recovery periods was between NoNo, on the one hand, and the HoHOX and NoHOX conditions, on the other (d = 0.93). In all cases, the TSI was lower after each bout of exercise than at baseline (Table 2) (d = 5.02).

**Table 2:** The values (means ± SD) of the EMG and the TSI responses of the athletes (n = 10) during the 3x3-min DP intervals in association with the different oxygen contents during the intervals and recovery periods. NoNo: normoxia with normoxic recovery; NoHOX: normoxia with hyperoxic recovery; HoHo: hypoxia with hypoxic recovery; HoHOX: hypoxia with hyperoxic recovery.

			**1** ^**st**^ **interval**		**2** ^**nd**^ **interval**		**3** ^**rd**^ **interval**	
**m. triceps brachii [%]**	**HoHo**		100±0.0		108.8±14.2		104.0±17.7	
	**HoHOX**		100±0.0		105.5±13.0		103.8±16.2	
	**NoNo**		100±0.0		115.1±13.9		116.1±24.9	
	**NoHOX**		100±0.0		108.2±11.1		102.3±19.3	
**m. biceps brachii [%]**	**HoHo**		100±0.0		102.6±22.6		112.7±39.3	
	**HoHOX**		100±0.0		137.6±98.2		123.6±45.9	
	**NoNo**		100±0.0		117.2±14.8		126.6±13.6	
	**NoHOX**		100±0.0		97.8±11.0		101.2±12.9	
**m. pectoralis major [%]**	**HoHo**		100±0.0		120.7±66.8		141.7±102.4	
	**HoHOX**		100±0.0		98.8±34.1		91.5±28.4	
	**NoNo**		100±0.0		109.2±20.4		116.4±32.6	
	**NoHOX**		100±0.0		153.8±152.3		109.4±55.2	
**m. latissimus dorsi [%]**	**HoHo**		100±0.0		131.5±68.6		106.1±18.1	
	**HoHOX**		100±0.0		106.7±12.9		103.1±14.0	
	**NoNo**		100±0.0		107.4±11.4		122.8±31.7	
	**NoHOX**		100±0.0		107.0±9.6		105.6±11.6	
		**baseline**	**1** ^**st**^ **interval**	**1** ^**st**^ **recov.**	**2** ^**nd**^ **interval**	**2** ^**nd**^ **recov.**	**3** ^**rd**^ **interval**	**3** ^**rd**^ **recov.**
**TSI [%]**	**HoHo**	100±0.0	82.1±5.7	97.3±7.7	81.2±7.5	99.6±3.1	80.9±7.2	99.7±3.2
	**HoHOX**	100±0.0	79.4±6.3	103.2±4.2	79.2±5.8	103.0±4.2^c^	78.7±6.0	103.1±4.1^c^
	**NoNo**	100±0.0	80.4±6.1	101.5±6.7	79.9±6.6	101.3±7.6^d^	80.6±7.1	99.1±6.2^d^
	**NoHOX**	100±0.0	79.3±8.9	101.9±1.8^c^	79.2±9.0	100.0±3.1	79.5±9.4	98.4±4.3

^c^ indicates significant difference to NoNo at the same time point

^d^ indicates significant difference to HoHOX at the same time point

After the warm-up under all four conditions, the blood lactate concentration was elevated (d = 2.34). In the case of HoHo the lactate concentration was higher before the second interval than for NoHOX and remained so until the end of the intervention (d = 1.0), except at the last time-point of measurement. At the same time, the pH fell continuously after the first interval until the last measurement under all four conditions (d = 4.08), most markedly during HoHOX (Table 1).

Table 3 depicts the mean power outputs (P_mean_) for the high-intensity intervals of all four interventions and Fig 1 shows the values in relation to the first interval. In HoHo the P_mean_ declined from the 1^st^ to the 3^rd^ interval (P < 0.05). In NoNo, NoHOX and HoHOX no decrements in performance were apparent for the relative values (best P < 0.94; best d = 0.26), whereas in the case of NoHOX and NoNo the absolute mean power was lower during the 3^rd^ than the first interval (P < 0.05).

**Table 3:** The mean (± SD) absolute power outputs in watt of the athletes (n = 10) during the three 3-min intervals of DP while breathing air of different oxygen contents during the intervals of exercise and recovery periods. NoNo: normoxia with normoxic recovery; NoHOX: normoxia with hyperoxic recovery; HoHo: hypoxia with hypoxic recovery; HoHOX: hypoxia with hyperoxic recovery.

	1^st^ interval	2^nd^ interval	3^rd^ interval
**HoHo**	220±29	195±28^a^	187±32^a^
**HoHOX**	215±28	198±26	189±26^a^
**NoNo**	218±27	203±26	200±26^a^
**NoHOX**	212±30	206±28	205±30

^a^ significantly different from baseline

## Discussion

Our primary aim here was to examine the influence of breathing different levels of oxygen during recovery on double-poling cross-country skiing at either sea-level or at simulated altitude of 1800 m. The major findings were as follows: 1) With hypoxic exercise, hyperoxic recovery elevated S_a_O_2_ to >99%. 2) TSI and iEMG were the same under all conditions. 3) Blood lactate levels with hypoxic exercise/hypoxic recovery were significantly higher than with normoxic exercise/hyperoxic recovery before the second session of double-poling and remained elevated until the end of the intervention (except at the last time-point of measurement). 4) With hyperoxic recovery following normoxic and hypoxic exercise, the athletes were able to maintain their P_mean_, whereas application of hypoxic recovery following hypoxic exercise led to a decline in P_mean_, but only during the third interval of high-intensity double-poling.

In a manner similar to other findings [10, 31], arterial oxygen saturation (S_a_O_2_) increased here to >99% during hyperoxic recovery following double-poling for 3 min under either normoxic or hypoxic conditions. In another study involving upper-body exercise, recovery of S_a_O_2_ was significantly faster with hyperoxic than with normoxic breathing [32]. In our case, S_a_O_2_ with hyperoxic breathing was already higher when blood was sampled immediately after exercise, perhaps due to the time required to sample capillary blood from the earlobe (approximately 15 s), during which the athletes were breathing heavily. Indeed, S_a_O_2_ is increased after as few as 25 breaths of hyperoxic gases (F_i_O_2_ = 0.60) [33]. The desaturation to 87.0% following mild hypoxic exercise and to 91.5% following normoxic exercise is comparable to other values reported after high-intensity exercise involving the upper [10, 32, 34] or lower body [2].

Although it has been shown that iEMG is altered by different F_i_O_2_, in the present investigation no differences in iEMG as potential indicator of muscular fatigue [35] between the four interventions were apparent. Central fatigue is characterized by a reduction in output of muscle force/power in response to the centrally mediated attenuation of motor drive and consequently lowered EMG activity [16, 35]. It is known, that muscle activity is influenced by the F_i_O_2_ of the air breathed [8, 18, 19], but we could not confirm these findings with our results.

Impairment of performance due to reduced neural drive to the active musculature occurs only under severe hypoxia as a result of stronger reflex inhibition by reduced brain oxygenation, independent of afferent feedback and peripheral fatigue [36], and/or a hypoxia-induced elevation in the levels of intramuscular metabolites known to stimulate muscle afferents [37]. Even though hypoxia augments the demands placed by exercise on the central nervous system, local metabolic factors may also contribute to the more pronounced fatigue. Here, the only metabolic factor we measured directly was blood lactate concentration, the results of which are in agreement with these speculations.

Furthermore, since the intensity of all the 3 x 3 min intervals was “all-out”, we can speculate that recruitment of muscle fibers was the same under all four experimental conditions. The enhanced availability of O_2_ to muscles during hyperoxia elevates peripheral metabolism and thereby lowers the extent to which activation of muscle fibers is necessary. Here, hyperoxia was applied only during the recovery periods. Perhaps the additional O_2_ during hyperoxic recovery was sufficient to allow muscle activity to remain unchanged during the intervals. Furthermore, the inhibitory feedback signals, triggered by accumulation of metabolites in working muscles, decreases neural drive and shift fatigue from peripheral to central processes that are not reflected in muscle activity [38].

As far as we know, this is the first report on oxygenation of the arm muscles (measured with NIRS) during high-intensity upper-body exercise under mild hypoxic conditions followed by hyperoxic recovery. Even though the muscle activation measured by EMG did not change within or between our interventions, the decrements in TSI from baseline and from the recovery periods to the end of the intervals of exercise were ~19–22%, which is comparable to the corresponding drop in the TSI of m. *triceps brachii* during a Wingate Anaerobic Test performed with the arms (22–34%) [39]. During anaerobic sprints with the legs, the reduction in TSI is much more pronounced. Here a ~80% drop following a Wingate test has been observed [39, 40]. During longer intervals (approximately 5 min) of ice speed-skating, the TSI of the m. *vastus lateralis* muscle decreases 15–22% [39]. Interestingly, the rate of desaturation in the arms during short (the Wingate test) and longer intervals of exercise (3 x 3 min) appears to be the same.

Furthermore, desaturation did not fall over the course of the three intervals here. During short, very high-intensity periods of exercise (e.g. the Wingate test), the intensive muscle contractions may restrict blood flow [40, 41], thereby reducing the oxygen supply. The arms possess a higher proportion of glycolytic muscles than the legs [42], and since O_2_ delivery to working glycolytic muscles is poorer than that to oxidative muscles, the reductions in TSI in the two sets of limbs are not comparable. These observations reveal that the arms work more anaerobically than the legs [43], producing relatively high power even with less oxygen, which explains why the drop in TSI during short sprints is not as high in the arms as in the legs. Furthermore, glycolytic fibers might benefit to a greater extent from the enhanced blood perfusion during sprints [11], helping to increase O_2_ delivery to the muscles and reduce the levels of inorganic phosphate and H^+^-ion [44].

We observed lower blood lactate concentrations after the first normoxic interval with hyperoxic recovery than under constantly hypoxic conditions. This enhanced clearance of blood lactate under hyperoxia had no effect on performance. Some authors have attributed the improved performance associated with hyperoxic breathing to attenuated accumulation of lactate in skeletal muscle and blood [45–47], perhaps reflecting slower net breakdown of glycogen during aerobic exercise [46–48]. However, findings with respect to arm exercises are discrepant with Sperlich and colleagues (2011) reporting the same blood lactate concentrations with hyperoxia and normoxia [34].

Hyperoxia does not appear to exert a beneficial effect on energy production during maximal-intensity exercise of short duration [34]. On the other hand, blood lactate concentrations were lower after three 3-min double-poling sprints with hyperoxic recovery [10]. It has been reported that due to the elevated oxidative capacity of their muscles, endurance-trained individuals profit more from the enhanced clearance of blood lactate during hyperoxic recovery after training [46]. Blood pH is also of importance in the context of recovery and performance. Adams and Welch showed that hyperoxic breathing during exercise alters pH significantly [49]. Since pH was lower only after the second interval of exercise under hypoxia-hyperoxia than normoxia-hyperoxia conditions, buffering was apparently not influenced by hyperoxia in the present study.

Besides the responses in the physiological parameters we could find an influence of hyperoxia and hypoxia on power output. Regarding the normalized power values (Fig 1), the athletes were able to maintain their P_mean_ with normoxic and hyperoxic recovery following normoxic and hypoxic exercise, whereas application of hypoxic recovery following hypoxic exercise led to a decline in P_mean_, but only during the third interval of high-intensity double-poling. Based on the absolute power values documented here, the athletes were only able to maintain their P_mean_ during the intervals of normoxia with hyperoxic recovery (Table 1). Only one previous study has demonstrated beneficial effects of hyperoxic breathing during recovery on performance [34]. The mean and peak power outputs of elite swimmers performing five 50-s intervals of arm exercise on a swim bench with 6-min intervals of hyperoxic recovery were 5% higher than with normoxic recovery [34]. These investigators suggested that the lower extraction of oxygen by arm than leg muscles, as observed in highly trained cross-country skiers [50], enhanced diffusion of oxygen to the mitochondria, resulting in greater arm power [34]. In that case the work-to-rest ratio was higher (~7:1) than in our present study (1:1) and therefore the hyperoxic recovery may have been more effective. In contrast, other reports have not revealed any beneficial effects of hyperoxic recovery on performance [10, 32].

## Conclusions

In the present study all of our athletes exhibited a significant decline in P_mean_ during the 3 x 3-min double poling sprints in hypoxia with hypoxic recovery. In addition, our findings indicate that when breathing normoxic, hyperoxic (F_i_O_2_ = 1.00) or hypoxic (F_i_O_2_ = 0.165) air during recovery from such sprints under normoxic or hypoxic conditions, the tissue saturation index (TSI) of the *m*. *triceps brachii* and the iEMG of various upper-body muscles are identical. Under normoxic and hypoxic conditions hyperoxic recovery prevents a decline in P_mean_, nor was there any apparent decline in NoNo; whereas application of hypoxic recovery following hypoxic exercise led to a decline in P_mean_. Therefore, we conclude that the less pronounced decline in P_mean_ during 3 x 3-min double-poling sprints in normoxia and hypoxia with hyperoxic recovery is not related to changes in muscle activity or oxygenation, but may aid athletes to recover from high intensity exercise. Potentially, the larger number of anaerobic muscle fibers in the arms might benefit to a greater extent from the elevated blood perfusion during these intervals, resulting in more delivery of O_2_ to arm muscles under hyperoxic conditions than during leg exercise.
